# Early access programs for medicines: comparative analysis among France, Italy, Spain, and UK and focus on the Italian case

**DOI:** 10.1186/s40545-023-00570-z

**Published:** 2023-05-17

**Authors:** Alice Tarantola, Monica Hildegard Otto, Patrizio Armeni, Francesco Costa, Francesco Malandrini, Claudio Jommi

**Affiliations:** 1 SDA Bocconi School of Management, Centre for Research on Health and Social Care Management (CERGAS), Milan, Italy; 2 Department of Social and Political Science, Bocconi University, Milan, Italy; 3 Department of Pharmaceutical Sciences, Università del Piemonte Orientale, Largo Guido Donegani, 2, 28100 Novara, Italy

**Keywords:** Early access programs, Off-label, Medicines, France, Italy, Spain, UK

## Abstract

Early access programs (EAPs) generally refer to patient access to medicines/indications before marketing authorization, possibly extended to price and reimbursement approval. These programs include compassionate use, which is usually covered by pharmaceutical companies, and EAPs reimbursed by third-party payers. This paper aims at comparing EAPs in four European countries (France, Italy, Spain, UK) and providing empirical evidence on EAPs in Italy. The comparative analysis was conducted through a literature review (including scientific and grey literature), complemented by 30-min semi-structured interviews with local experts. The Italian empirical analysis employed data available on the National Medicines Agency website. Although EAPs are very different across countries, they exhibit some common features: (i) eligibility criteria refer to the absence of valid therapeutic alternatives and a presumed favourable risk–benefit profile; (ii) payers do not allocate a pre-determined budget to these programs; (iii) total spending on EAPs is unknown. The French EAPs seem to be the most structured, financed through social insurance, covering pre-marketing, post-marketing and pre-reimbursement phases and providing for data collection. Italy’s approach to EAPs has been varied, with several programs covered by different payers, including the cohort-based 648 List (for both early access and off-label use), the nominal-based 5% Fund, and Compassionate Use. Most applications to EAPs are from the Antineoplastic and immunomodulating drug class (ATC L). Some 62% of indications in the 648 List are either not under clinical development or have never been approved (pure off-label use). For those subsequently approved, most approved indications coincide with those covered through EAPs. Only the 5% Fund provides data on economic impact (€ 81.2 million in 2021; average cost per patient € 61.5K). Diverse EAPs are a possible source of inequalities in access to medicines across Europe. A harmonization of these programs, though difficult to achieve, could be modelled on the French EAPs and provide key advantages, not least of which a common effort to collect real-world data in parallel with clinical trials and clear separation between EAPs and off-label use programs.

**Supplementary Information:**

The online version contains supplementary material available at 10.1186/s40545-023-00570-z.

## Background

In the European Union (EU) there are two ways to marketing authorization (MA) of new drugs: a centralized process through the European Medicines Agency (EMA) and a national procedure (managed by national drug agencies) [1–4]. While MA is a precondition, it is not the only causal factor of market access in Europe [3]. Pricing and reimbursement (P&R) negotiation, which occurs at the national level, represents the second step of the access process [4]. P&R is complex to manage due to large variability in procedures, timelines, and criteria in each country’s P&R decision-making process [3, 5]. Hence, the approval process may not always achieve an appropriate balance in providing rapid access to promising new drugs, ensuring safety and efficacy, and meeting subsequent evidence requirements for Health Technology Assessment (HTA) bodies and payers concerning relative effectiveness and cost-effectiveness compared with therapeutic alternatives [6].

Different programs have emerged to accelerate access to medicines. The EU has introduced several programs to accelerate the development and approval of medicines, including the PRIME scheme to support the development of medicines that target an unmet medical need [1]. Other programs allow early access (early access programs, or EAPs) to an unlicensed drug under development (drugs before MA), or off-label use, the intentional prescribing of an authorized drug for an unapproved indication which is not under development [7, 8]. In all these programs the costs are covered by the National Healthcare System with the exception of the Compassionate Use Program that is a treatment option that allows the use of an unauthorized medicine; it involves direct and free delivery of the medicine by the manufacturer [1].

EAPs typically cover the period before MA but can be extended to the period between approval and the national P&R decision [3].

EMA has recommended that EU countries include within EAPs patients who have been treated in a clinical trial and who wish to continue the treatment [1], despite the worldwide recognition that patients treated in a clinical trial have the option of continuing treatment for an extended period of time in an open-label extension study aimed at generating long-term data on the efficacy, safety, tolerability, and administration of the drug [9, 10].

Nevertheless, EAPs have mostly been introduced at the country level. France can be considered a pioneer, the first to create a legal framework in 1994 [11]. In 2020, the French Ministry of Health proposed and introduced important reforms of the Temporary Authorization Program (ATU) system [3]. In the UK, the Early Access to Medicines Scheme (EAMS) was introduced for medicines targeted at life-threatening or seriously debilitating conditions and a clear unmet need [3].

Italy has instead managed its EAPs over time through ad hoc regulations: Law 648/96 and Law 326/96—the 5% Fund [12]. The 648 List includes both cohort-based early access and off-label use. The 5% Fund covers orphan drugs and medicines in development for rare and severe diseases not yet approved.

The existing literature has investigated the main eligibility criteria for EAPs and their benefits and risks. Eligibility criteria include an expected positive benefit–risk balance (i.e., the potential benefit to public health of the drug’s immediate availability outweighs the potential risks associated with the greater level of uncertainty), that comprehensive confirmatory clinical data will be provided within a reasonable timeframe, and that the drug addresses an unmet medical need [2, 3, 13].

A number of studies have addressed the benefits and risks of EAPs [11, 14, 15]. Indeed, these programs allow patients with rare and/or severe diseases to be treated with potentially life-saving drugs in an ethical and compliant manner [15]. They represent a fundamental part of a company’s global market access plans and strategies [15] and can be helpful in early market penetration, this is of paramount importance even after the commercial launch of the product for its leading to increased acceptance and uptake by physicians and patients [15]. On the other side, early access decisions are particularly challenging due to the limited clinical evidence on a drug’s benefit–risk profile and uncertainty regarding the cost-effectiveness of the relevant medicines [3].

A comparative analysis of EAPs in major European countries is lacking, that includes eligibility criteria, program duration, decision-making processes, impact on P&R negotiation, and data on overall impact. In addition, no empirical evidence is available on the impact of EAPs in Italy, with the exception of an estimate of the economic impact of compassionate use programs [16].

This paper aims to fill this literature gap, comparing EAPs in major European countries and providing an in-depth analysis of Italian EAPs.

## Methods

### International comparative analysis

The comparative analysis was carried out through an extensive literature review complemented by semi-structured interviews with local experts. France, Italy, Spain, and the United Kingdom (UK) were considered among the largest European countries. Germany was not included, since we were not aware of any EAP, most likely because the market access process is very swift once a drug has been approved [5].

The first step was to search Pubmed, Google Scholar, and Scopus to retrieve articles published in the last 3 years. References included in these articles and published in previous years were also scrutinized where fitting with the topic of interest.

The following search terms were used in combination with each country (France, Italy, Spain, and the UK): “Early access” or “Early Access Program(s)” or “Off-label” or “Expanded access” or “Foreign access” and “Drugs” or “Medicines” or “Pharmaceuticals”. Specific terms were used for France (“ATU”), Italy (648 Law, 5% Fund, well-established use), and for the UK (“Early Access Medicine Schemes”). Data were extracted using country-specific search terms from different fields of study, such as health policies, medicines for rare diseases, and healthcare access.

Data sourcing was not limited to articles published in indexed journals, but extended to include grey literature:

- Government websites of respective countries: HAS (Haute Autorité de Santé, the French HTA body for scientific and cost-effectiveness assessment. https://www.has-sante.fr) and ANSM (Agence Nationale de Sécurité du Médicament et des Produits de Santé. https://ansm.sante.fr) for France; AIFA (Agenzia Italiana del Farmaco. https://www.aifa.gov.it) for Italy; AEMPS (Agencia Espanola de Medicamentos y Productos Sanitarios. https://www.aemps.gob.es) for Spain; and NICE (the National Institute for Health and Care Excellence. https://www.nice.org.uk), MHRA (the Medicine and Healthcare products Regulatory Agency https://www.gov.uk/government/organisations/medicines-and-healthcare-products-regulatory-agency) and NHS (National Health Service. https://www.nhs.uk) for the UK;
- the European authority’s website: EMA (the European Medicines Agency. https://www.ema.europa.eu/en);
- other websites found by googling the search terms for each country (listed above): Cancer Research UK (https://www.cancerresearchuk.org), Remap Consulting UK (https://remapconsulting.com), BlueReg Pharma Consulting (https://blue-reg.com), Omedit (https://www.omedit-idf.fr), Insideeulifesciences (https://www.insideeulifesciences.com), and blogs (Simon-Kucher.com and gd-associes.com).

The literature review was complemented by open-ended interviews lasting 30 min on average with local experts. The interviews were conducted using Teams, they were recorded, and the transcripts were validated by the experts, three interviews were carried out for Spain, and two for the UK. Only one, mainly confirmatory, interview was conducted for France, since the literature on this country is quite complete. As for Italy, we have chosen not to carry out interviews, since we deemed that desk research on the Italian context (both grey and peer-reviewed), along with our personal expertise, was sufficient to obtain a comprehensive overview of the Italian framework. Additional file 3: Appendix 3 illustrates the questions raised to the experts.

Data retrieved from the literature and the interviews were comparatively organized into eleven items:

- Regulatory reference;
- Type of disease and drugs included in the program;
- Type of program: cohort EAP vs. nominal EAP;
- Procedures, application, and management of the program;
- Maximum duration foreseen;
- Evidence required (eligibility criteria);
- Pricing of interested medicines;
- Existence and management of reporting activity;
- Existence of Managed Entry Agreements (MEA);
- Effects on pricing after MA;
- Availability of data on the allocated budget and the budget spent by third-party payers.

### Programs in Italy

Data on EAP in Italy for each of the relevant programs (648 List, Well-established Use List, and the 5% Fund List—see below) were found on the AIFA website (https://www.aifa.gov.it/en/web/guest/home). Information on the approved and reimbursed indication was retrieved from the website of the National Archive of Official Gazettes (https://www.gazzettaufficiale.it), the official journal of record of the Italian government.

Regarding medicines/indications currently inserted in the 648 list (last update: 02/2022), we retrieved the:

- Regulatory reference;
- Indication for which the inclusion in the 648 list was requested;
- Anatomical Therapeutic Chemical (ATC) code;
- Date of inclusion;
- Reason for the inclusion request in the 648 List;
- Negotiated price and/or the availability of information on the expected cost per patient;
- Eventual MEA with the inclusion in the 648 List [17];
- MA and difference, if any, between the 648 List indication and the approved indication;
- Reimbursement status and difference, if any, between the reimbursed indication and the approved indication;
- Reimbursement status:
  - Innovativeness status;
  - Reimbursement class: class A, which includes medicines reimbursed in both retail and hospital markets (essential drugs and drugs for chronic diseases and class H, which includes drugs reimbursed only in the hospital setting;
  - Ex-factory price;
  - (hidden) discount;
  - MEA;
  - Other market access requirements (e.g., prescription limited to specific health centers/specialists; drug registry).

Data for early access covered through the 5% Fund were retrieved from AIFA’s website (https://www.aifa.gov.it/en/web/guest/home, latest update considered: 04/2022. The relevant document includes the applicant (healthcare center, department, and physician) and the authorized expenditure. Since the ATC class was not explicitly listed, the name of the requesting department was used to determine ATC classification. In some cases, identifying the anatomic category (first letter in the ATC class, see the list of abbreviations section) was impossible. Therefore, we created two additional classes, "Uncertain” and “Not identified”. “Uncertain class” includes drugs that may belong to multiple categories. For instance, applications from immunology departments were impossible to classify into a single anatomic category (molecules used to treat these pathologies can belong to several classes, L, B, or I). The "not identified” class includes drugs for which the requesting department was not indicated and thus we could not associate an ATC class with the medicine.

AIFA publishes disease-specific lists of well-established uses of medicines (these drugs are administered to patients for an unauthorized prescription on the basis of their consolidated use supported by the literature), with a distinction between adult and pediatric indications. For each molecule, the authorization request for clinical practice and clinical evidence supporting the request are indicated. The anatomic category was identified for each molecule.

The list (downloaded in April 2022) is divided into the following categories:

- Adult-use:
  - Cancer drugs (latest update: 01/2022),
  - Hematological drugs (latest update: 06/2021),
  - Neurological drugs (latest update: 01/2022),
  - Medications after transplant (latest update: 07/2019),
  - Radiopharmaceuticals (latest update: 10/2021),
  - Antiviral drugs (latest update: 12/2014),
  - Cardiovascular drugs (latest update: 01/2019),
  - End of life and palliative medicines (latest update: 11/2018);
- Pediatric use:
  - Cancer drugs (latest update: 07/2020);
  - Cardiovascular drugs (latest update: not available);
  - Anti-infectives (latest update: 03/2020);
  - Anaesthetics (latest update: 04/2020);
  - Gastrointestinal medicines (latest update: 04/2020);
  - Hematopoietic medicines (latest update: 01/2019);
  - Dermatology medicines (latest update: 07/2012);
  - Genito-urinary disorders and sex hormones (latest update: 07/2021);
  - Central nervous system and skeletal muscle medicines (latest update: 07/2019);
  - Respiratory drugs (latest update: 07/2012);
  - End-of-life and palliative medicines (latest update: 11/2018).

## Results

### International comparative analysis

Our cross-country analysis shows that EAPs are embedded in the general framework of the special use of drugs. This includes: (i) EAP strictu sensu, that is, early access to drugs under development (before MA); and (ii) off-label use. The former includes compassionate use programs (generally covered by the pharmaceutical industry), access to medicines approved in other countries but not yet available, and early access programs covered by third-party payers.

Our focus is on early access programs covered by third-party payers, but the distinction between compassionate use and EAP covered by third-party payers is not always straightforward.

EAPs exhibit some common characteristics as well as important differences across countries, as shown in the profiles in Table 1 (and the application process described in Additional file 1: Appendix 1). Common characteristics include a highly formalized application process, not necessarily embedded in a legal framework. Each country’s healthcare system, including drug agencies, healthcare organizations, and physicians, is systematically involved in the management of these programs (application, assessment, use), whereas the role played by HTA authorities and industry varies across countries. Eligibility criteria include evidence on the efficacy and safety profile, target disease (severe, rare, or disabling disease), absence of (valid) alternatives, and a convincing claim that the treatment is not deferrable.

**Table 1:** EAP: comparison among the major European Countries

EAP	France	Italy	Spain	UK
General Label	Early access (ex-ATU and ex-PEC-T) + Others	Early access and off-label use	Availability of medicines under special circumstances	EAMS (Early Access to Medicine Schemes)
Regulatory reference	LOI n° 2020-1576 du 14 décembre 2020 de financement de la sécurité sociale pour 2021	648 List: Law 648/96, Law 79/2014, Decreto 2/8/2019 5% Fund: Law 326/03	Royal Decree 1015/2009 (under review)	No regulatory references so far
Named/Cohort	Named or cohort depending on the program	648 List: cohort 5% Fund: named	Named	Both: named/cohort
Coverage	Before the completion of the P&R process	Before MA	Foreign medicines: after MA Off-label: no MA	Only before MA
*Process*				
Applicant	Industry/physicians—hospitals	648 List: Patients Associations, Scientific Societies, Health Care Organization, Universities, Clinicians 5% Fund: Health Care Organization (specialised centres), Clinicians	Physicians/hospitals	Industry/physicians—hospitals
Approval	ANSM (safety and efficacy) HAS (other selection criteria)	AIFA (CTS)	AEMPS; Regional authorities (in special occasions for expensive drugs)	MHRA
Payers of medicines	Hospitals (after receiving an internal permit) But companies could provide medicine for free	648 List: Regions 5% Fund: AIFA through 5% Fund fed by the pharma industry	Hospitals via Regions/industries (most compassionate use cases)	Industries (for this reason is not an EAP as we intend)
*Selection criteria*				
Target diseases	Severe, rare or disabling diseases, no alternatives,	648 List: Different types of disease 5% Fund: rare diseases and particular/severe diseases	Severe diseases, no alternatives	Severe and disabling disease, high unmet need
Medicines	New drugs/ indications in development, off-label drugs, foreign medicines	648 List: No valid alternatives; cheaper than valid alternatives 5% Fund: Orphans drug/drugs in development not approved yet, which represent "a hope of therapy"	Off-label medicines, Foreign medicines	New Drugs, products already marketed in the UK for other indications (off-label), foreign medicines
Evidence	Safety, efficacy (based on the results of clinical trials) and presumed to be innovative	648 List: Phase II/Data that may support their use (pure 'off-label') 5% Fund: Clinical report	Safety and efficacy	Safety and efficacy and presumed to be innovative
Data collection	Yes (well structured). PUT -RD (patient characteristics, medicine usage, efficacy, quality of life (PROMs etc.…), adverse events	648 List: in principle data on efficacy and safety profile (from the regions to AIFA) Data are not available 5% Fund: No	Yes. Physicians have to collect data on adverse events/ AEMPS has no obbligation to collect data, but may collect them	No evidence on data availability
Data on economic impact	No	Yes (but only for the 5% Fund)	No	No
Dedicated Fund	No	Only for 5% Fund	No	No
MEA	Financial-based agreements (volumes caps/payback if the awarded price < price charged through early access programs)	No, except in 648 List where they are rarely applied	No	No
Impact on P&R	Data collection influences ASMR’ evaluation and, therefore, the price of the drug	648 List (revenue from 648 are considered in the P&R negotiation)	No	No
Fee	No	No	No	Yes
Limited duration time and, if any, renewal rules	Duration of early access cannot exceed 1 year. It can be renewed with updated product information. If the HAS opinion is negative, the company is obliged to provide patients with treatment for 1 year before stopping supply	No	No	Scientific opinion could be renewed at least 2 months before expiry

France has a long tradition of EAPs through its ATU. EAPs have recently undergone reform through the *LOI 2020–1576 du 14 décembre 2020 de financement de la sécuritè sociale pour 2021*, which has formally introduced a program known as Accès précoce. This program concerns indications under development, extended through completion of P&R negotiation (AP2—*Accès Précoce 2*, Early Access after Marketing Authorization), and is covered by the social insurance system but without a dedicated fund.

Formally, the duration of early access cannot exceed 1 year, but it can be renewed with updated product information. The program continues until price negotiation is completed and the price is published. However, discounts over list prices can be applied if 90 days of price negotiation with CEPS (*Comité Economique des Produits de Santé*, Economic Committee for Health Products) after obtaining HAS evaluation. If HAS does not recommend the reimbursement, the company is forced to provide patients with treatment for 1 year before stopping the supply.

Besides the above-mentioned eligibility criteria, the presumption of innovativeness is considered. To meet the definition of innovative, a medicine should:

- bring a substantial change to patients, in terms of efficacy, safety, convenience of use, and organizational impact;
- be accompanied by a development plan and clinical results supporting the presumption of benefits for the patient;
- not have any important unknown factor relating to tolerance or other important data.

Manufacturers apply to ANSM, the regulatory body in charge of safety and efficacy assessment. After ANSM approval, the request needs to be approved by HAS.

Once the manufacturer receives authorization (which should take a maximum of 3 months after HAS’s analysis), if it is a new medicine it can freely set the drug price for the whole EAP period (*Indemnitè*, if the product is not provided for free). If the object of the EAP is a new indication, the price already set for previously approved indications is applied. *Accès précoce* contracts provide for two financial managed agreements. Volume caps are set, and if they are exceeded the difference is covered by the company. If the price ultimately negotiated through the P&R process is lower than the one set for the EAP, the difference is paid back by the company. EAPs include both named patient requests (nominal) and cohort-based programs.

Data collection, through the PUT-RD (*Protocole d’Utilisation Thérapeutique*) protocol, is a relevant part of French EAPs and is quite extensive, including patient characteristics, condition of use, safety, and efficacy (primary endpoint, mortality data, etc.), with both clinical and patient-reported outcomes (adverse events, quality of life). Data are collected by healthcare professionals, shared with industry, and sent to HAS. Data can be used to support the product value proposition when it is appraised for reimbursement.

In Italy**,** there are several EAPs: the most important are the compassionate use (DM 8/5/2013, amended by DM 7/9/2017), the 648 List (introduced with Law 648/1996 and amended by Law 79/2014 and *Decreto* 2/8/2019), the Well-established Use List, and the AIFA National Fund (Law 326/2003—“5%” Fund).

Compassionate use covers medicines/indications for which there are no valid therapeutic alternatives. These medicines/indications can be under clinical development or approved but not yet covered by the National Health Service. Medicines used in compassionate use programs are fully covered by pharmaceutical companies. These programs can be nominal and cohort-based and should be approved by the hospital Ethics Committee; the approval of cohort-based programs should be notified to AIFA.

The 648 List includes both cohort-based early access and off-label use. Drugs for diseases without valid alternatives (access issues) or that are less expensive than available therapies (economic issues) may be listed in the 648 Program. Medicines approved under the 648 List program are fully covered by the National Health Service. AIFA’s Technical Scientific Committee (CTS) approves applications for inclusion in the 648 List. Many stakeholders (patient associations, scientific societies, health care organizations, clinicians), with the exception of the industry, can apply for the 648 List. In principle, the Italian regions should send a report on the clinical and economic impact of the drugs included in the 648 List to AIFA on a quarterly basis. In reality, no data are systematically collected and publicly available.

The 5% Fund covers orphan drugs and medicines in development for rare and severe diseases, not yet approved. The fund is managed by AIFA and financed by a 5% tax on commercial expenses paid by all pharmaceutical companies. The application for the 5% Fund is nominal, managed by physicians or health care organizations, and approved by the AIFA’s CTS.

In Spain, EAPs are regulated by Royal Decree 1015/2009 (currently under review) and known as “Availability of medicine under special circumstances”. Despite the existence of specific legislation, EAPs for medicines are not very structured. The programs are all nominal-based with the exception of compassionate use, which could be also cohort-based.

In contrast to other countries, manufacturers do not submit applications (and do not pay fees). The request is sent by physicians/hospitals to the AEMPS for approval and to contact the company to discern the product’s availability. In certain circumstances, Regional Authorities can discuss the use of a particularly expensive drug in a forum (the Permanent Pharmacy Commission).

Medicines used in EAPs are in principle covered by the health care system until MA and, similar to France, the manufacturer freely sets the price, usually equal to the price already set for other indications for already approved and reimbursed medicines. However, in the case of early access, pharmaceutical companies normally cover the cost of the drug, similar to the British example. Charging the price to the health care system is more frequent for off-label use. In this case hospitals will pay for the required drugs, after receiving regional authorization in the form of an internal permit.

There are no data collection requirements, though the AEMPS may collect data in special circumstances. In all cases, these data are not considered for P&R negotiation.

At present in Spain, there is no EAP similar to the Italian 5% Fund or cohort-based 648/96. Compassionate use could be charged to hospitals, but this happens very rarely.

EAPs in the UK, known as EAMS, cover drugs before MA, and include all patients eligible for clinical trials. EAMS can be nominal or cohort-based. In principle, the authorized manufacturer provides the drug for free after receiving the hospital's request. Since companies bear all costs, these schemes are comparable to compassionate use in Italy. EAMS are regulated by administrative documents which have so far not been translated into legislation.

MHRA analyzes the data sent by the manufacturer. Once a drug is considered safe, effective, and innovative in treating a particular disease, MHRA notifies NICE in England (or the Scottish Medicines Consortium in Scotland) and the NHS. To be included in the EAMS a medicine should obtain a Promising Innovative Medicine (PIM) designation and a positive Scientific Opinion (based on the benefit–risk profile of the medicine provided by the company).

For PIM designation, the drug must meet four criteria:

- the condition should be life-threatening or seriously debilitating: the severity of the disease should be justified based on objective and quantifiable medical or epidemiologic information, in terms of mortality and morbidity, with special emphasis on patient quality of life;
- the unmet need for the relevant indication should be high, i.e., no method of treatment, diagnosis or prevention is available, or existing methods have serious limitations;
- the medicinal product is likely to offer a major advantage over methods currently used in the UK: preliminary evidence based on non-clinical and clinical data should indicate that the advantage and magnitude of the effect claimed for the product is predicted to be of significant relevance to the patient and will address their unmet need;
- the potential adverse effects of the medicinal product are likely to be outweighed by the benefits, allowing for the reasonable expectation of a positive benefit–risk balance.

The manufacturers pay a fee for each of these steps.

EAMS is valid for 1 year, can be renewed, and expires with the MA. There is no evidence of how data collected through EAMS might affect the HTA process managed by NICE.

### Programs in Italy

Our empirical analysis of Italian EAPs focuses on Law 648/96 (648 Program), Well-established Use, and the 5% Fund. Additional file 2: Appendix 2 provides a comparative analysis of these programs along with compassionate use.

Data on these programs are not fully comparable. Applications to the 648 List and the Well-Established Use Lists refer to the whole cohort and the number of involved patients is not available, whereas the 5% Fund requests are made at the patient level.

**Fig. 1:**
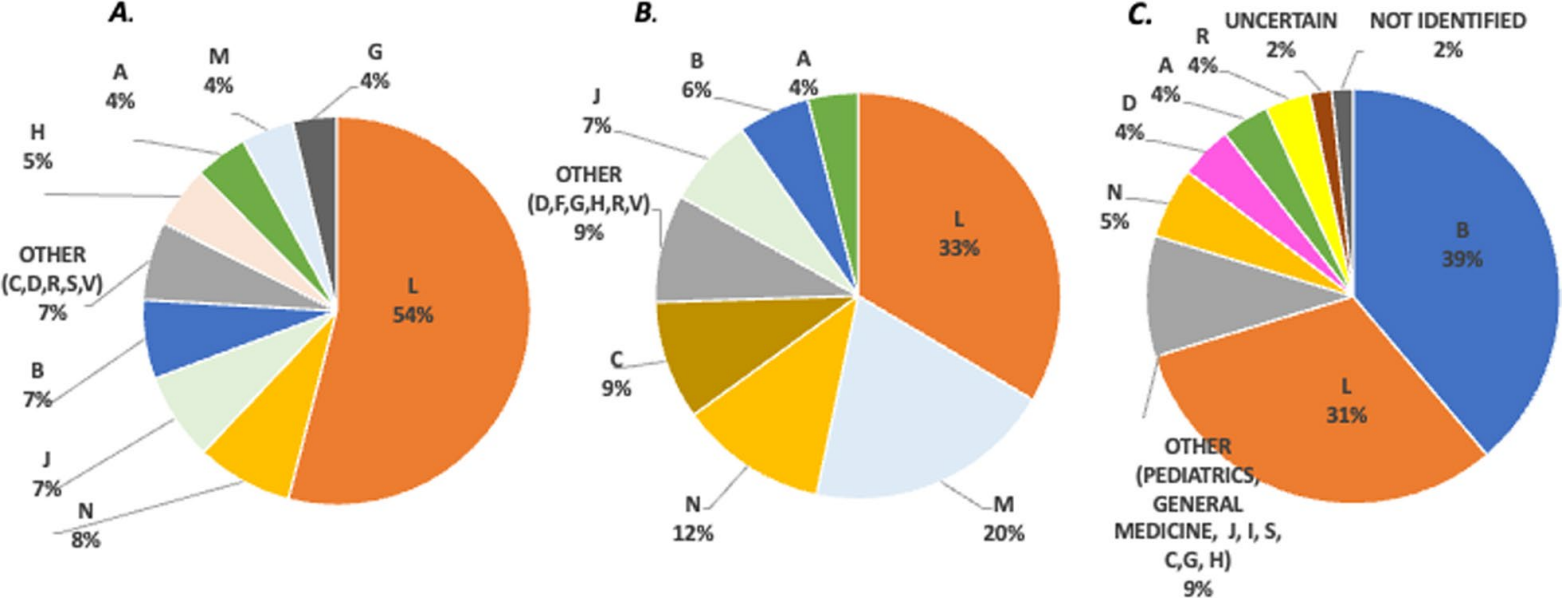
EAP in Italy: ATC class distribution. **A** 648 list, **B** well-established use, **C** 5% fund

In general, most applications to EAPs belong to the Antineoplastic and immunomodulating agents class (ATC L) (Fig. 1).

In detail, 97 molecules/indications are included in the 648 List, 54% of the total molecules belong to ATC L class, followed by ATC N (Nervous System), 7% represent classes J (Anti-infectives for systemic use) and B (blood and blood-forming organs), respectively. There are 289 molecules listed in the Well-established Use programs, for both adult use (126 submissions) and pediatric use (164 submissions). ATC L molecules account for 33% of the total, followed by Musculoskeletal system diseases (ATC M—20%), and Nervous System disorders, ATC N (12%). The 5% Fund accounted for 1805 applications, mostly belonging to the blood and blood-forming organ group, ATC B (39% of the total), followed by Antineoplastic and immunomodulating agents, ATC L (31% of the total), and drugs used for Nervous System disorders, ATC N (5%), Fig. 1A*.*

AIFA’s website provides some additional data on the 648 List and the 5% Fund. With the exception of bevacizumab, which has been included in the 648 List for economic reasons, in all other cases medicines have been listed in the 648 Program due to the absence of valid therapeutic alternatives. The price charged to the SSN (*Sistema Sanitario Nazionale*, the Italian NHS) is not mentioned for 85% of indications included in the 648 List. This is due to the high frequency of drugs joining this program through an extension of indications that are already approved and reimbursed.

**Fig. 2:**
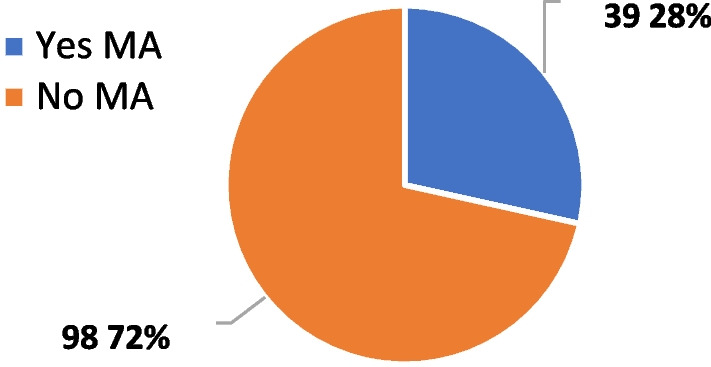
Marketing authorization (MA) for drugs listed in the 648 list

**Fig. 3:**
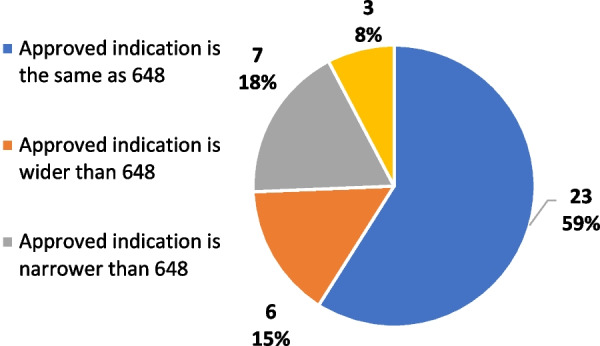
Approved vs. 648 list indication

As mentioned before, the 648 List concerns both off-label and early access use. Only 38% of the indications included in the 648 List have achieved European MA (Fig. 2). In most cases (59%) the approved indication overlaps with the indication covered through early access, whereas for 15% and 18% of cases subsequent approval has extended or restricted, respectively, the indication (Fig. 3).

Among the 648 List indications that received MA, 49% are reimbursed (typically for the same approved indication), while 51% are not yet covered, because either the relevant manufacturer has not yet applied to AIFA or P&R negotiation is underway or, in a minority of cases, the drug is not reimbursed.

**Fig. 4:**
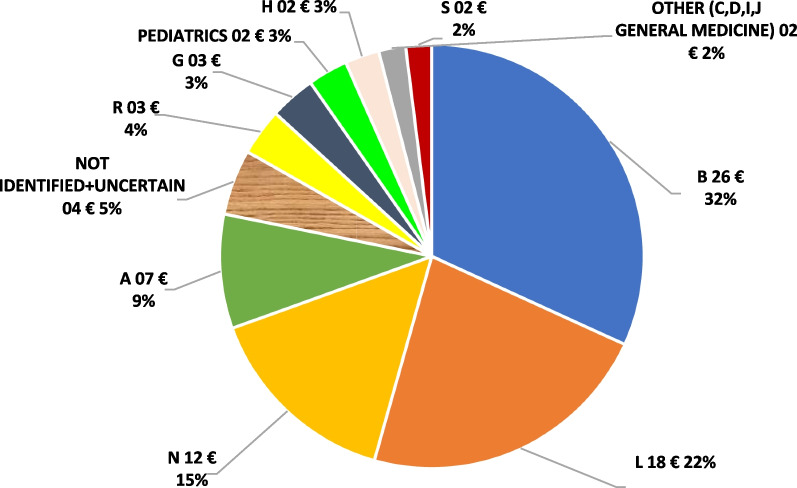
5% fund: total expenditure (in millions) per ATC class

Data on costs sustained for EAPs are available only for the 5% Fund. Total expenditure in 2021 amounted to € 81.2 million. Expenditure by ATC class (Fig. 4) reflects the applications (Fig. 1C). Class B total expenditure amounts to € 25.9 million which corresponds to 32% of total requests, followed by class L (€ 18.4 million—22% of the total), and class N (€ 12.3 per million—15% of the total).

**Fig. 5:**
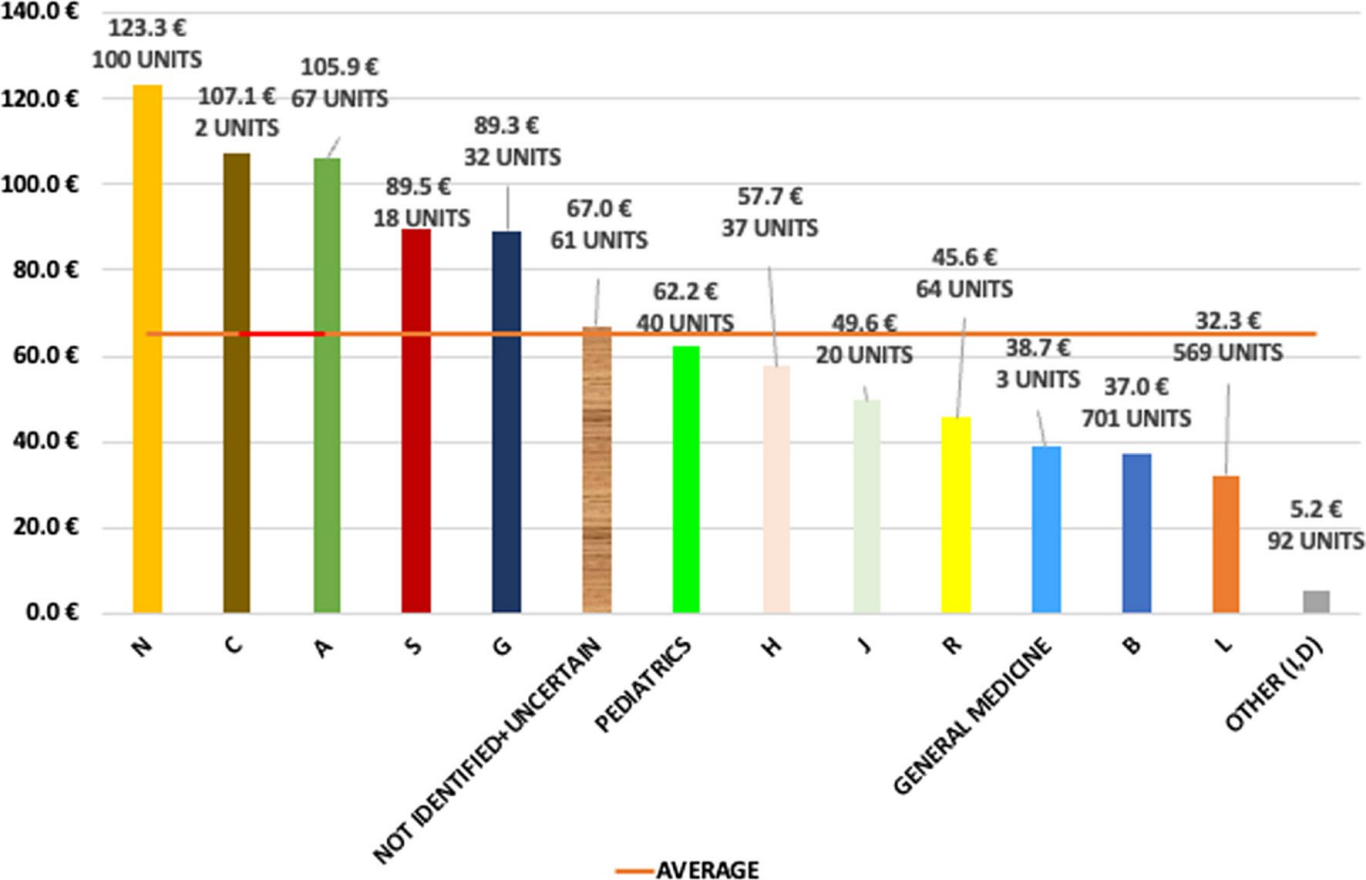
5% fund: unitary cost (in 1000 s) per each ATC class

The average unitary cost is € 61.5K. Drugs in class N are the most expensive (unitary cost equal to € 123.3K), followed by class C (unitary cost equal to €107.1K per two units) and class A (€105.9K per 67 units) (Fig. 5).

## Discussion

Our paper has investigated EAPs for medicines in several major European countries, with in-depth analysis of the Italian case.

We found some common features among programs. An unmet need and a presumed favorable risk–benefit profile are common among selection criteria. Third-party payers, if any, do not allocate a pre-determined budget to these programs: volumes caps are contracted with the relevant companies only in France. EAP total spending is unknown, with the exception of the 5% Fund in Italy, with the stated purpose of nominal access to orphan drugs for severe diseases.

However, there are more differences than similarities among these programs. These differences concern who pays for EAPs, the period of coverage, and formal requirements for data collection. The French EAP is the most advanced, financed through social insurance, covering both the pre-marketing and post-marketing (and pre-reimbursement) phases and providing for data collection. Italy has implemented different programs covered by different payers. Early access is extended to the post-marketing (and pre-reimbursement) phase. Data collection is formally required by some programs, but has not been implemented so far. In Spain and the UK, EAPs coincide with compassionate use, which are mostly covered by industry, limited to the pre marketing phase, and provide for data collection only on adverse events.

The most important cohort-based EAP in Italy is the 648 List. The program was originally designed for off-label use of indications that are not under a clinical development process by the producing pharmaceutical company. Off-label use is allowed in the case of high unmet need and provision of sufficient evidence on the medicine’s risk–benefit profile. However, the application process was recently extended to include indications, where medicines used off-label are cheaper than drugs already approved for the same indication. This has provided the 648 List with a new guise as a cost-containment policy. The role of the 648 List has been gradually extended to include early access for medicines under development, thus introducing a hybrid nature in the program, though off-label use still outstrips early access. Antineoplastic and immunomodulatos are most medicines more often included in the 648 List. The economic impact of the 648 List is unknown, but available for the 5% Fund, which provides for early nominal access to medicines for rare and severe diseases. Applications for the 5% Fund are approved and covered by AIFA, but the fund is financed by the pharmaceutical industry, whereas 648 List programs are fully covered by the SSN).

To the best of our knowledge, our study is the first comparative analysis of EAP in major European countries. In addition, it provides interesting insight into critical issues related to these programs, including the general absence of a pre-defined budget and the hybrid nature of these programs in some jurisdictions (in particular, in Italy) that may create confusion regarding their main objectives. Furthermore, data collection, despite formally required, has not been implemented. This may lead to the impression that EAPs are implemented only to accelerate patient access to medicines and not to collect information that may complement evidence from clinical trials,

The main limitation of our study is that it represents a purely descriptive analysis relying on the existing literature, interviews with experts and secondary data. We did not perform a perceptual analysis of pros and cons of these programs, which would require a larger expert panel and analysis of the current regulations. This is recommended for future analyses.

## Conclusions

Our paper highlighted that there are important differences in EAPs implemented by the largest European countries. France seems to have the most advanced EAPs. Early access to medicines is highly regulated and covered by social insurance. Coverage lasts until price negotiation is finalised, and financial entry agreements are contracted with the relevant companies to avoid an excessive burden for third-party payers. EAPs are clearly distinguished from off-label access to indications that are not under a clinical development process. Applications to early access are appraised on the grounds of structured parameters that include, among other criteria shared by all countries, the presumption of innovativeness. The new EAP approved in 2021 requires the collection of data that can be integrated with evidence from clinical trials.

Italy has been quite generous in implementing EAPs, compared to other countries under investigation (the UK and Spain). However, too many initiatives have been implemented and the most important cohort-based program (648 List) has mixed early access with off-label use and prioritization of access for clinical need with off-label use for economic reasons. Furthermore, pharmaceutical companies are not allowed to apply for these programs. This is quite unusual for medicines in development. Finally, data collection is formally envisaged but not implemented.

There are four key lessons from a policy viewpoint.

The first is that different EAPs across countries introduce another source of inequity in access to medicines in Europe, besides differing times to access and reimbursement rates. With a more coordinated action, equity would be improved. Once the programs are implemented, they could be partially or fully covered by third-party payers or industry depending on the ability to pay. Adoption elsewhere of EAPs modelled on the French program would facilitate a common effort to collect real world data that may be integrated with evidence from clinical trials. These data might then support medicine assessment and appraisal processes at both the European (Joint Clinical Assessment within the new HTA regulation) and country levels.

The implementation of an EAP modelled on the French program would be easier in Italy, since it has a longer tradition on EAP. The application of an EAP similar to the French one in Italy would require (i) allowing industry to directly apply to an EAP, (ii) applying the present criteria used in Italy for the evaluations of innovativeness (unmet need, added therapeutic value and quality of the evidence); implementing data collection systems.

The third is that EAP in Italy should be clearly distinguished from the off-label use programs, whereas the 648 List includes both medicines. That is, EAPs should be clearly aimed at providing early access, possibly reimbursed by third-party payers, to medicines in development which are presumed to generate important value for patients and the health care system. Off-label programs, in turn, should cover indications for medicines which are not under development, but with some evidence on their risk–benefit profile. Furthermore, the economic arguments, that are also a driver of off label use, could be also considered, but not at the expenses of evidence which is required to achieve reimbursed off-label status.

Finally, the financial impact of these programs should be carefully monitored and the risk of excessive burden could be offset by implementing financial-based agreements.

## Supplementary Information

**Additional file 1**:** Appendix 1**. EAP process in France, Italy, Spain, and UK.

**Additional file 2**:** Appendix 2**. EAP in Italy, including Compassionate use.

**Additional file 3**:** Appendix 3**. Questions raised to the experts.

## Abbreviations

AAC: *Autorisation d’accès compassionnel.* Compassionate Use Authorisation
AAP: *Autorisation d’accès précoce.* Early Access Authorisation
AEMPS: *Agencia Española de Medicamentos y Productos. Sanitarios* Spanish Medicines Agency
AIFA: *Agenzia Italiana del Farmaco*. Italian Medicines Agency
ANSM: *Agence Nationale de sécurité du Médicament et des produits de santè.* French Medicines Agenc
AP 1/2: *Accès Précoce** 1/2*. Early Access 1/2
ATC: Anatomical Therapeutic Chemical
ATC A: Alimentary tract and metabolism
ATC B: Blood and blood-forming organs
ATC C: Cardiovascular system
ATC D: Dermatologicals
ATC G: Genito urinary system and sex hormones
ATC H: Systemic hormonal preparations, excl. sex hormones and insulins
ATC J: Antiinfectives for systemic use
ATC L: Antineoplastic and immunomodulating agents
ATC M: Musculo-skeletal system
ATC N: Nervous system
ATC P: Antiparasitic products, insecticides, and repellents
ATC R: Respiratory system
ATC S: Sensory organs
ATC V: Various
ATU: *Autorisations Temporaires d'Utilisation.* Temporary Use Authorization
CEPS: *Comité économique des produits de santé.* Economic Committee for Health Products
CPC: *Cadre de prescription compassionnel.* Compassionate Prescribing Framework
EAMS: Early Access to Medicine Schemes
EAP: Early Access Programs
HAS: *Haute Autoritè de Santé*, French National Authority for Health
HCP: Health Care Provider(s)
HTA: Health Technology Assessment
MA: Marketing Authorization
MEA: Managed Entry Agreement
MHRA: Medicines and Healthcare products Regulatory Agency
NICE: National Institute for Health and Care Excellence
P&R: Price and Reimbursement
PIM: Promising Innovative Medicine
PUT-RD: *Protocole d’Utilisation Thérapeutique**–**Recueil de Données*. Data Collection Protocol
SO: Scientific Opinion
SSN: *Servizio Sanitario Nazionale*, The Italian NHS

## References

[1] https://www.ema.europa.eu/en/human-regulatory/research-development/compassionate-use. European Medicines Agency (EMA). https://www.EmaEuropaEu/En/Human-Regulatory/Research-Development/Compassionate-Use n.d.

[2] Pignatti F, Gravanis I, Herold R, Vamvakas S, Jonsson B, Marty M (2011). The European Medicines Agency: an overview of its mission, responsibilities, and recent initiatives in cancer drug regulation. Clin Cancer Res.

[3] Martinalbo J, Bowen D, Camarero J, Chapelin M, Démolis P, Foggi P (2016). Early market access of cancer drugs in the EU. Ann Oncol.

[4] Kempf E, Zalcman G, Lebbe C (2021). National early access programs and clinical trials: what opportunities for early access to therapeutic innovations for patients with malignant melanoma?. Cancer.

[5] IQVIA. EFPIA Patients W.A.I.T. Indicator 2021 Survey - Updated July 2022. https://www.EfpiaEu/Media/676539/Efpia-Patient-Wait-Indicator_update-July-2022_finalPdf n.d.

[6] Eichler H-G, Abadie E, Breckenridge A, Flamion B, Gustafsson LL, Leufkens H (2011). Bridging the efficacy–effectiveness gap: a regulator’s perspective on addressing variability of drug response. Nat Rev Drug Discov.

[7] Iudicello A, Alberghini L, Benini G, Mosconi P (2016). Expanded Access Programme: Looking for a common definition. Trials.

[8] Urbinati D, Toumi M (2012). PHP147 Early Access Programmes (EAPs): review of the European System. Value in Health.

[9] Taylor GJ, Wainwright P (2005). Open label extension studies: research or marketing?. BMJ.

[10] Richard Chin BL. Principles and Practice of Clinical Trail Medicine. 1° Edition. 2008.

[11] Early Access Programs in Europe: A Regulatory Tool with Pre‐marketing Impact‐the Pharma Letter. http://www.ThepharmaletterCom/Article/Early‐access‐Programs‐in‐europe‐a‐regulatory‐tool‐with‐pre‐marketing‐Impact. Accessed 30 Apr 2015; 2008.

[12] Apolone G, Ardizzoni A, Buzzetti G, Clerico MA, Conte P, de Braud F, De Lorenzo F, Ferrandina MG, Genazzani A, Gori S, Maio M, Patroncini M, Perrone F, Scambia G, Scroccaro G (2019). Early access in oncology: why is it needed?. Glob Region Health Technol Assess..

[13] The European Medicines Agency (EMA) EA to M. https://www.ema.europa.eu/en/documents/leaflet/early-access-medicines-development-support-regulatory-tools_en.pdf. Accessed 24 Nov 2022.

[14] Simon E (2015). Integrating managed access programs: global considerations. Appl Clin Trials..

[15] Patil S (2016). Early access programs: benefits, challenges, and key considerations for successful implementation. Perspect Clin Res.

[16] Jommi C, Pantellini F, Stagi L, Verykiou M, Cavazza M (2021). The economic impact of compassionate use of medicines. BMC Health Serv Res.

[17] Jommi C, Armeni P, Costa F, Alberti C, Bandello F, Bordonaro R, Caprodossi A, Di Maio M, Gaudioso A, Giuliani G, Langella R, Marata AM, Patarnello F, Pinto C, Rasi G, Villa F (2021). Programmi di early access dei farmaci e managed entry agreement in Italia: i risultati di un Focus Group (programmi di early access e managed entry agreement) [Early access programs and managed entry agreements for medicines in Italy: results of a Focus Group (Early Access Programs and Managed Entry Agreement).]. Recent Prog Med.

