# Supplementary figures and images for: Early access programs for medicines: comparative analysis among France, Italy, Spain, and UK and focus on the Italian case
Source: J Pharm Policy Pract. 2023 May 17;16:67. doi: 10.1186/s40545-023-00570-z (PMC10193685; doi:10.1186/s40545-023-00570-z)

## APPENDIX 1

### EAP process in France, Italy, Spain, and UK

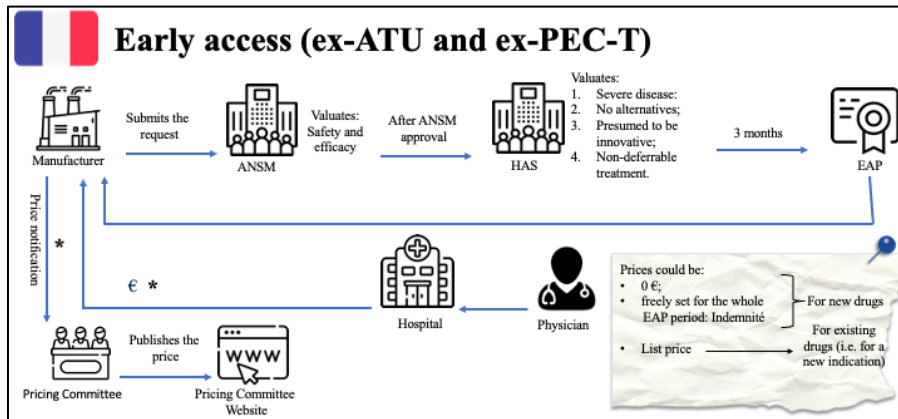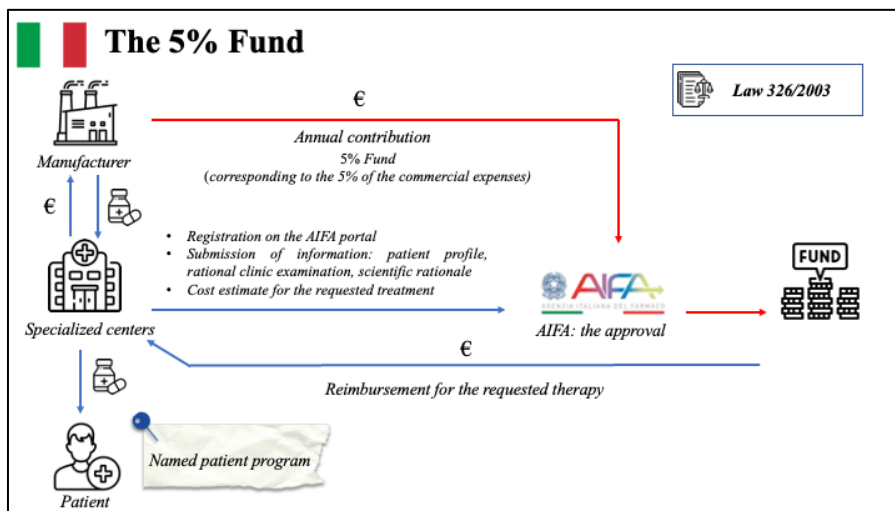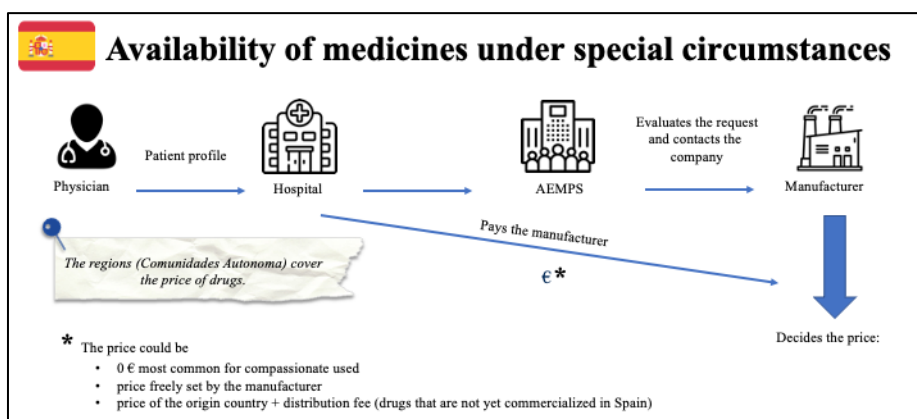

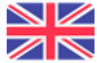

## EAMS (Early Access to Medicine Schemes)

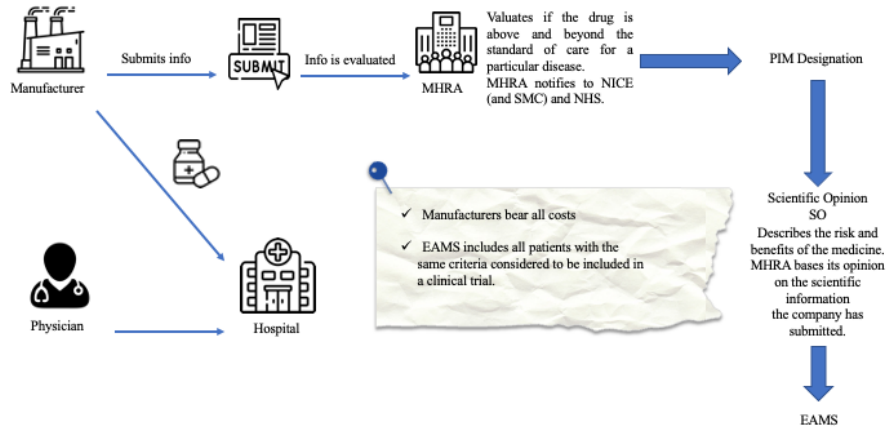

Supplement: Supplementary file 1 — Additional file 1: Appendix 1. EAP process in France, Italy, Spain, and UK. [file 40545_2023_570_MOESM1_ESM.pdf]
