# Supplementary material for: Early access programs for medicines: comparative analysis among France, Italy, Spain, and UK and focus on the Italian case
Source: J Pharm Policy Pract. 2023 May 17;16:67. doi: 10.1186/s40545-023-00570-z (PMC10193685; doi:10.1186/s40545-023-00570-z)
Supplement: Supplementary file 2 — Additional file 2: Appendix 2. EAP in Italy, including Compassionate use. [file 40545_2023_570_MOESM2_ESM.pdf]

APPENDIX 2

EAP in Italy, including Compassionate use

EAP		Compassionate use	648 List	5% Fund
General Label		Accesso precoce e uso off label		
Regulatory Reference		DM 8/5/2013 - DM 7/9/2017	Legge 648/96, Legge 79/2014, Decreto 2/8/2019	Legge 326/03
Named / Cohort		Named / Cohort (expanded access)	Cohort	Named
Coverage		Before MA / Till the medicine is 'available'	Before MA (but expanded for new drug till the completion of P&R process)	Before MA (after MA for orphan drugs)
Process	Applicant	Clinicians	Patient Associations, Scientific Societies, Health Care Organisations, IRCCS, University, Clinicians, AIFA (CTS)	Reference / Specialised Centres (online form till July 2022)
	Approval	Ethics Committee (Notification to Aifa for cohort-based)	Aifa (CTS): simplified price negotiation (Decreto 2/8/2019)	Aifa (CTS)
	Payers of medicines	Industry	Regions	AIFA through 5% Fund fed by the industry
Selection criteria	Target diseases	Severe diseases or rare life-threatening diseases	-	Rare diseases / Particular and severe diseases
	Medicines	No valid alternatives<br>Medicines / Indication under clinical development<br>Medicines / Indications with MA but not available yet	No valid alternatives<br>Cheaper than valid alternatives	Orphan drugs / Drugs in development not approved yet "which represent a hope of therapy"
	Evidence	Phase II (Phase I per rare diseases and rare tumours)	Phase II / Data that may supporto their use (pure 'off label')	Clinical report
Data collection		Adverse reactions (to notify to AIFA)	In principle data on efficacy and safety profile (from the Regions to AIFA) (data are not available)	No
Data on economic impact		-*	In principle data on efficacy and safety profile (from the Regions to AIFA) (data are not available)	Till 2017 dedicated fund and consolidated data on applications. 2020 only dedicated fund. From 2020 data on each single application
Dedicated budget		-	No	Yes (funded by the industry)
MEA		No	Rarely applied	No
Impact on P&R		?	Yes (medicines in 648 as comparators / revenue from 648 considered in P&R negotiation)	?
