# Supplementary material for: Early access programs for medicines: comparative analysis among France, Italy, Spain, and UK and focus on the Italian case
Source: J Pharm Policy Pract. 2023 May 17;16:67. doi: 10.1186/s40545-023-00570-z (PMC10193685; doi:10.1186/s40545-023-00570-z)
Supplement: Supplementary file 3 — Additional file 3: Appendix 3. Questions raised to the experts. [file 40545_2023_570_MOESM3_ESM.pdf]

APPENDIX 3

List of questions about EAP in France

1. Is there any dedicated fund by the social health insurance system for EAP? How is this fund determined? Do you know the fund for 2022?
2. In France, EAP can be activated after Market Authorization (AP2) for new patients. Is it correct?
6. What roles do ANSM and HAS play in the AAP program (Early Access Authorization)? We understand that ANSM evaluates efficacy and safety, while HAS plays other roles (e.g. it evaluates the presumed innovation). Is it correct? Do manufacturers pay fees for each step of the *Autorisation d'accès précoce* (AAP) program? Which is the amount of these fees per step?
7. Monitoring and data collection: apart from the exception provided for *Cadre de prescription compassionel* (CPC) and *Autorisation d'accès compassionel* (AAC), is data collection the same for AAP and CPC/AAC? Is there any other data collection? How is these data used? Does the manufacturer finance data collection? Could you provide a complete picture of data collection within EAP? We understand that Health Care Providers (HCP) (and patients for the quality of life data) should complete 4 forms: initiation of therapy, follow-up, cessation of therapy, and quality of life evaluation. Is it correct?
8. Who does determine the price of medicines in AAP programs? Is the pharma company free to set prices for medicines in AAP programs?
9. Is there any evidence of the economic impact of AAC/AAP?
10. Is evidence collected during the EAP (e.g. impact of expenditure, outcomes, etc.) used during the pricing and reimbursement negotiation?
11. Are other Managed Entry Agreements (MEAs) associated with EAP, beyond rebates based on sales amount (i.e. other financial-based MEAs or outcome-based MEAs)?
12. EAP and Pricing and reimbursement. What does "free of charge" mean? What is the meaning of "maximum fee" applied by the manufacturer? We understand there are two rebates: the first is applied every year (depending on volumes for the indication covered by the AAP), while the second rebate is applied retroactively (it is equal to the difference between the negotiated price with the CEPS and the AAP's final price). Is the second rebate applied as a payback?

List of questions about EAP in Spain

1. Is there an EAP for medicines (excluding compassionate use), i.e. a program that is covered by the Spanish National Health Service before the medicine / indication gets the marketing authorization? Is this program extended to the phase between marketing authorization and inclusion into the reimbursement list?
2. Is this program managed at the central level? Is there any regional initiative?
3. Can you provide us the regulatory references apart from Royal Decree 1015/2009?
4. Is it both a nominal and cohort program?
5. Which are the inclusion/exclusion criteria? Which criteria are considered for off label drugs?
6. Who does apply for EAP? Who does evaluate and approve the application? Which evidence are required? Are fees applied to applicants? How is the price determined? Who does pay (Central Government, Regional Authorities, single hospitals)?
7. Are EAP renewable? Is a maximum duration foreseen for EAP?
8. Is there any obligation to collect data on effectiveness, adverse effects, cost, …?
9. Is there any dedicated fund for EAP?
10. What is the economic impact of the EAP in Spain?
11. Do EAP programs influence the pricing and reimbursement process?
12. Are MEA (Managed Entry Agreement) associated with EAP? Which kind of MEA? Who defines them?

List of questions about EAP in UK

1. Could you explain the role of the EAMS plus? Can new patients be recruited after MA?
2. Can EAMS be renewed after MA?
3. Is there any evidence of the economic impact of EAMS on the National Health Service?
4. Do EAMS programs influence the pricing and reimbursement process?
5. Is there any obligation to collect data on effectiveness, adverse effects, cost …? Is data collected actually?
6. We understand that EAMS were introduced in 2014, but we lack of the relevant legislation. Was the regulatory framework amended afterward?
7. Since the EAP is renewable, is there a maximum allowable duration of the EAP in the UK?
8. How are NICE and National Health Service involved in the EAP's first step (PIM Designation)?
9. What is the necessary level of therapeutic value to get the PIM Designation? Is the economic impact considered as well to get PIM? Which are the criteria to get the PIM Designation?
10. Does awarding a PIM designation imply automatically the inclusion in EAMS? Which criteria are considered to get a positive EA scientific opinion? Once you get the PIM designation and the

company decides to go for EAMS, then what are the criteria that are most important for getting a positive early access SO?

11. How are the drug prices for EAMS determined?
12. Are data collected through EAMS used in the following Technology Appraisal?
13. Are MEAs associated with EAMS? Which kind of MEAs? Who defines them?
14. Who is responsible for defining what risk-sharing agreement and what can be combined?
